# Suppression of Human T Cell Proliferation Mediated by the Cathepsin B Inhibitor, z-FA-FMK Is Due to Oxidative Stress

**DOI:** 10.1371/journal.pone.0123711

**Published:** 2015-04-27

**Authors:** Tanuja Rajah, Sek Chuen Chow

**Affiliations:** School of Science, Monash University Sunway Campus, Jalan Lagoon Selatan, Bandar Sunway, 46150, Selangor Darul Ehsan, Malaysia; University Freiburg, GERMANY

## Abstract

The cathepsin B inhibitor, benzyloxycarbonyl-phenylalanine-alanine-fluoromethyl ketone (z-FA-FMK) readily inhibits anti-CD3-induced human T cell proliferation, whereas the analogue benzyloxycarbonyl-phenylalanine-alanine-diazomethyl ketone (z-FA-DMK) had no effect. In contrast, benzyloxycarbonyl-phenylalanine-alanine-chloromethyl ketone (z-FA-CMK) was toxic. The inhibition of T cell proliferation mediated by z-FA-FMK requires not only the FMK moiety, but also the benzyloxycarbonyl group at the N-terminal, suggesting some degree of specificity in z-FA-FMK-induced inhibition of primary T cell proliferation. We showed that z-FA-FMK treatment leads to a decrease in intracellular glutathione (GSH) with a concomitant increase in reactive oxygen species (ROS) levels in activated T cells. The inhibition of anti-CD3-induced T cell proliferation mediated by z-FA-FMK was abolished by the presence of low molecular weight thiols such as GSH, N-acetylcysteine (NAC) and L-cysteine, whereas D-cysteine which cannot be metabolised to GSH has no effect. The inhibition of anti-CD3-induced up-regulation of CD25 and CD69 expression mediated by z-FA-FMK was also attenuated in the presence of exogenous GSH. Similar to cell proliferation, GSH, NAC and L-cysteine but not D-cysteine, completely restored the processing of caspase-8 and caspase-3 to their respective subunits in z-FA-FMK-treated activated T cells. Our collective results demonstrated that the inhibition of T cell activation and proliferation mediated by z-FA-FMK is due to oxidative stress via the depletion of GSH.

## Introduction

Halomethylketone peptides such as peptidyl chloromethylketones were the first active site directed irreversible enzyme inhibitors synthesised and were originally designed as potential drugs for the treatment of certain diseases [1,2]. However, the highly electrophilic chloromethylketone moiety was too reactive and results in the alkylation of non-target molecules indiscriminately [3,4]. Efforts to replace the reactive chlorine atom led to the eventual synthesis of peptidyl fluoromethylketones [3]. Because of the much stronger carbon-fluorine bonds relative to carbon-chlorine bonds, fluoromethylketones were expected to be poorer alkylating agents and should reduce the non-specific alkylation significantly compared to chloromethylketones. However, once synthesised, peptidyl fluoromethylketones were found to be highly reactive and are selective irreversible inhibitors for cysteine proteases [4].

Benzyloxycarbonyl-phenylalanine-alanine-fluoromethylketone (z-FA-FMK) was originally designed as an affinity label to irreversibly block cathepsin B, a cysteine protease [3,4]. It was found to bind tightly to the enzyme active site and became a very potent inhibitor of cathepsin B. The enzyme is normally found in the lysosomes of cells, but in rheumatoid arthritis (RA) patients the enzyme activity of cathepsin B was found to be increased in the synovial fluid and synovial lining [5,6]. This suggests that cathepsin B may be a good target for therapeutic intervention for the treatment of RA using z-FA-FMK. Indeed, in vivo studies demonstrate that z-FA-FMK was extremely efficient in preventing the destruction of articular cartilage and bone in chronic inflammatory arthritis induced by adjuvant in mice [7–9]. However, accumulating evidences suggest that the remarkable therapeutic action of z-FA-FMK in the treatment of RA observed in mice may not be due to the inhibition of cathepsin B alone. Previous study has shown that z-FA-FMK inhibits LPS-induced cytokine secretion in macrophages by blocking the transactivation potential of NF-ĸB [10]. We have shown that besides blocking cathepsin B activity, z-FA-FMK effectively blocked human T cell activation and proliferation in vitro, and modulates host response to pneumococcal infection in vivo [11]. The inhibition of human T cell activation and proliferation mediated by z-FA-FMK was accompanied by the blocking of the activation of caspase-8 and caspase-3 [11]. Although caspases play a pivotal role in apoptosis, it is now established that caspases such as caspase-8 play an important role in T cell activation and proliferation and that blocking the activation of this enzyme will ultimately block T cell activation and proliferation [12,13]. Taken together, these studies suggest that the pleiotropic immunosuppressive effects of z-FA-FMK may account for the remarkable therapeutic effect in suppressing articular cartilage and bone destruction in chronic inflammatory arthritis in mice [7–9].

In the present study, we examined the effects of other z-FA-FMK analogues such as z-FA-DMK and z-FA-CMK on T cell activation and proliferation. Our results showed that z-FA-DMK has no effect on T cell proliferation whereas z-FA-CMK was toxic to primary T cells. The immunosuppression mediated by z-FA-FMK is dependent on the FMK group and the benzyloxycarbonyl group at the N-terminal. We observed that z-FA-FMK treatment leads to depletion of intracellular GSH level in anti-CD3-stimulated primary T cells with a concomitant increase in reactive oxygen species (ROS) level. The inhibition of anti-CD3-induced T cell proliferation mediated by z-FA-FMK was abolished by low molecular weight thiols such as NAC, GSH and L-cysteine but not with D-cysteine. Taken together, these results suggest that z-FA-FMK-mediated inhibition of T cell proliferation is due to oxidative stress via the depletion of intracellular GSH.

## Materials and Methods

### Reagents

The following chemicals were obtained from Sigma Aldrich (USA): Glutathione (GSH), L-cysteine, D-cysteine, N-acetylcysteine (NAC), L-Buthionine-(S,R)-sulfoximine (BSO), monochlorobimane (MCB) and dihydroethidium (DHE). Monoclonal antibody (mAb) against CD3 (clone OKT3) was purified from hybridoma (ATCC) culture supernatants. Lymphoprep was from Axis-Shield PoCAS (Norway) while RPMI 1640 and FCS were from Gibco (UK). FITC-conjugated anti-CD25 and PE-conjugated anti-CD69 were acquired from BD Pharmingen (UK). The 5-bromo-2'-deoxyuridine (BrdU) labelling kit was obtained from Roche (Switzerland). Rabbit antibodies to caspase-3, mouse antibodies to β–actin and goat antibodies to caspase-8 were all from Santa Cruz Biotechnology (USA). All secondary HRP-conjugated antibodies were purchased from Dako (UK). Benzyloxycarbonyl-phenylalanine-alanine-fluoromethylketone (z-FA-FMK), benzyloxycarbonyl-tyrosine-valine-alanine-aspartic acid- fluoromethylketone (z-YVAD-FMK), benzyloxycarbonyl-valine-arginine-proline-DL-arginine-fluoromethylketone (z-VRPR-FMK) and benzyloxycarbonyl-phenylalanine-alanine-diazomethylketone (z-FA-DMK) were purchased from Bachem (Switzerland). Benzyloxycarbonyl-phenylalanine-alanine-chloromethylketone (z-FA-CMK) and biotinylated-phenylalanine-alanine-fluoromethylketone (b-FA-FMK) were from MP Biomedicals (USA).

### Peripheral mononuclear blood isolation

Peripheral venous blood was obtained from normal healthy volunteers. Each blood donor was individually informed and gave his/her consent for using the collected blood samples in a scientific study. Collection and use of blood samples in this study was approved by Monash University Human Research Ethics Committee (Reference Number: CF09/1065-2009000486). Human peripheral blood mononuclear cells (PBMCs) were separated from the red blood cells using density gradient centrifugation. In brief, the peripheral blood was diluted with RPMI and layered onto lymphoprep (density gradient of 1.077) and centrifuged at 800 x g for 30 min. The PBMCs were then removed from the interface between the lymphoprep and the plasma, washed and re-suspended in RPMI containing 10% (v/v) foetal calf serum (FCS), 10 mM L-glutamine (Invitrogen, UK), penicillin (100 U/ml) and streptomycin (100 μg/ml) until used. The viability of PBMCs was assessed using trypan blue exclusion assay and routinely determined to be >95%.

### Cell proliferation assays

The proliferation of T cells following anti-CD3 stimulation was determined using a colorimetric immunoassay based on the measurement of BrdU during DNA synthesis (Roche, Switzerland). The BrdU assay was performed according to the manufacturer’s instructions. In brief, PBMCs were seeded at 1 x 10^6^ cells/ml in RPMI 1640 supplemented with 10% FCS and stimulated with plate-bound 5 μg/ml anti-CD3 in the absence or presence of various peptidyl halomethylketones in an atmosphere of 5% CO_2_ in air at 37°C. The cells were cultured for various time periods with the last 3 h pulsed with 10 μM BrdU per well. At the end of the culture period, the plates were centrifuged and cells fixed with 200 μl ethanol (70%) in HCl (final concentration 0.5 M) for 30 min at -20°C. Following fixation, the DNA in the cells was partially digested by nuclease treatment for 30 min at 37°C before incubating with a horse radish peroxidase-conjugated BrdU antibody. After three rounds of washing, a substrate was added and the coloured product was measured after 20 min incubation (room temperature) at 405 nm with a reference wavelength of 495 nm using a microplate reader (Tecan 200).

### Determination of intracellular GSH in activated T cells

The intracellular GSH level in activated T cells was determined as described previously [14–17]. Following treatments, the cells (1 x 10^4^ cells) were centrifuged down at 3500 rpm for 10 min and washed with 100 μl PBS. The supernatant was carefully removed before adding 100 μl of 100 μM MCB (in PBS) for 30 min at 37°C in the dark. Unbound MCB is almost nonfluorescent, whereas the dye fluoresces blue when bound to GSH. The fluorescence in the samples was determined using a Tecan Infinite M200 fluoro-plate reader with excitation and emission wavelengths of 390 and 460 nm, respectively. A control containing media alone plus MCB was used as a blank and subtracted from the sample absorbance.

### Detection of ROS in activated T cells

Intracellular ROS in activated T cells was detected by using the redox sensitive fluorescent dye, DHE. Once inside the cells DHE will be oxidised by the ROS to form ethidium which binds to the nuclear DNA and emits a red fluorescence that can be detected with a flow cytometer. Following treatments, cells (1 x 10^6^) were washed and the cell pellet re-suspended in 1 ml of pre-warmed serum-free RPMI. DHE was added to a final concentration of 5 mM and the cells were incubated in the dark for 30 min at 37°C, washed with ice-cold PBS before re-suspending in 1 ml of PBS prior to analysis by flow cytometry. The samples were gated to include 1 x 10^4^ small resting T cells and large activated T cells, excluding cell debris, based on the forward- and side-scatter profiles. For the fluorescent 2-hydroxyethidium, an excitation wavelength of 532 nm (FL- 2 channel) was used and the machine was calibrated using unstained cells prior to each experiment.

### Determination of cell surface CD25 and CD69 expression using flow cytometry

Following treatments, PBMCs (1 x 10^6^) were centrifuged down and the supernatants discarded. The cell pellets were washed in ice-cold PBS and re-suspended in staining buffer (50 μl PBS containing 2% BSA). Fluorochrome-conjugated antibody (anti-CD25-FITC or anti-CD69-PE) in a final dilution of 1:50 was added to the cells and incubated on ice for 30 min in the dark. The cells were then washed twice in staining buffer before analysis using flow cytometry. Excitation wavelengths of 488 nm (FL-1 channel) (anti-CD25-FITC) and 532 nm (FL-2 channel) (anti-CD69-PE) were used. The machine was calibrated using unstained cells prior to each experiment.

### Western Blotting

Following treatments, the PBMCs were layered over lymphoprep and centrifuged to remove the dead cells. The viable cells were washed in PBS and re-suspended in an appropriate volume (10 μl per 1 x 10^6^ cells) of lysis buffer (0.1 M NaCl, 1 mM Tris HCl at pH7.6, 1 mM EDTA, 1% Triton-X, 1 mM PMSF). The cells in lysis buffer were taken through 3x freeze/thaw cycles using liquid nitrogen. Protein concentration was measured using the Bradford assay (Biorad, Germany). Protein equivalent to 20 μg whole-cell lysates was diluted in loading buffer (2% SDS, 10% Glycerol, 50 mM Tris-HCl pH 6.8, 0.2% Bromophenol Blue and 100 mM DTT) and resolved using 13% SDS-polyacrylamide gel electrophoresis. The separated proteins were transfer onto Hybond C membrane (Amersham, UK) and probed with antibodies to caspase-8 and caspase-3. Detection was carried out using chemiluminescence (Amersham). Following caspase detection, the membrane was incubated with stripping buffer before re-probing with antibodies to β-actin.

### Statistical analysis of the data

The experimental data were analysed using Student’s t test or One-way analysis of variance followed by Dunnet’s test.

## Results

### The role of the FMK moiety in z-FA-FMK-induced inhibition of anti-CD3-mediated T cell proliferation

**Fig 1:**
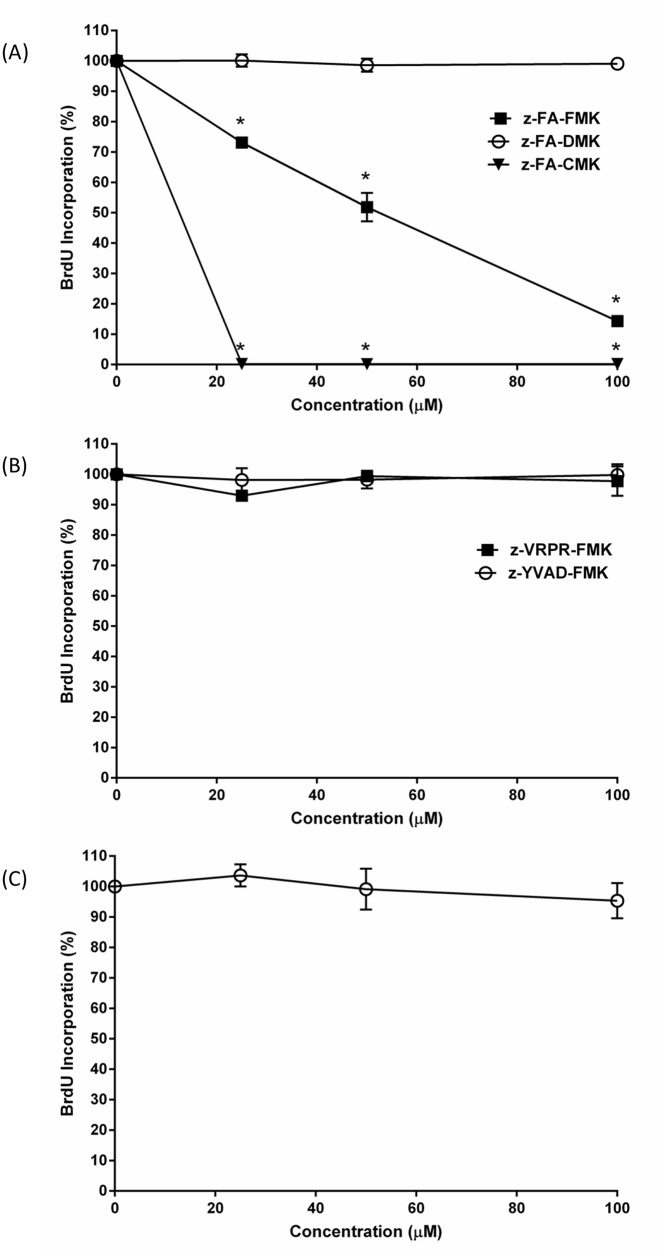
Effect of z-FA-FMK and other peptidyl methylketones on primary T cell proliferation. PBMCs (1 x 10^6^/ml) were stimulated with anti-CD3 (5 μg/ml) in the presence of various concentrations of z-FA-FMK analogues (A), different peptidyl FMKs (B), or different N-terminal groups (C) for 72 h. T cell proliferation was determined using BrdU incorporation as outlined in Materials and Methods. The relative incorporation of BrdU into control proliferation T cells (with anti-CD3 only) were normalised to 100%. The results are the means ± SEM from three separate donors. *,Significantly decreased (p < 0.01) from control.

We have previously shown that the cathepsin B inhibitor, z-FA-FMK was immunosuppressive in vitro and in vivo [11]. To further understand the mechanism that underlies the immunosuppression mediated by z-FA-FMK, we first examined whether the FMK group plays a role in suppressing T cell proliferation by comparing it with two of its analogues, z-FA-DMK and z-FA-CMK. As illustrated in Fig 1A, T cell proliferation induced by anti-CD3 was inhibited in a concentration-dependent manner by z-FA-FMK (IC_50_ ~50 μM), which is very much in line with our previous report [11]. However, when the FMK group was replaced with the DMK group, z-FA-DMK up to 100 μM had no inhibitory effect on anti-CD3-induced T cell proliferation. In sharp contrast, when the FMK group was replaced with the CMK group, z-FA-CMK completely abolished T cell proliferation induced by anti-CD3. Complete inhibition of T cell proliferation was apparent in the presence of 25 μM z-FA-CMK, suggesting that this methylketone peptide may be toxic as reported by us recently in Jurkat T cells, a leukemic T cell line [18]. Indeed, z-FA-CMK-treated cells were dead after 24 h as determined by PI uptake (data not shown). Since only z-FA-FMK blocked T cell activation and proliferation, the results suggest that the FMK moiety plays an important role in the immunosuppressive properties of this peptidyl halomethylketone. However, two other FMK containing methylketone peptides, z-YVAD-FMK, a caspase-1 inhibitor, and z-VRPR-FMK, a MALT1 inhibitor, were found to have no effect on anti-CD3-induced T cell proliferation (Fig 1B). This suggests that the FMK group per se is not causing the immunosuppression and that the inhibition of T cell proliferation mediated by z-FA-FMK involves the combination of the FMK moiety and other parts of the molecule. We next examined whether the benzyloxycarbonyl (z) group at the N-terminal of z-FA-FMK play any role in blocking T cell activation and proliferation. To this end, biotin-FA-FMK, which has a biotin molecule at the N-terminal instead of the benzyloxycarbonyl group, was examined. As illustrated in Fig 1C, biotin-FA-FMK up to 100 μM was unable to block T cell proliferation after 72 h. This lack of inhibition is unlikely to be due to biotin-FA-FMK not getting into the cells since avidin-FITC readily labelled the biotin-FA-FMK inside the cells indicating that the peptide is permeable and readily enters the cells (results not shown). Taken together, these results suggest that the benzyloxycarbonyl group at the N-terminal of the peptidyl methylketone also plays a role in the immunosuppressive effects of z-FA-FMK.

### Effect of z-FA-FMK on intracellular GSH and ROS levels in primary T cells

**Fig 2:**
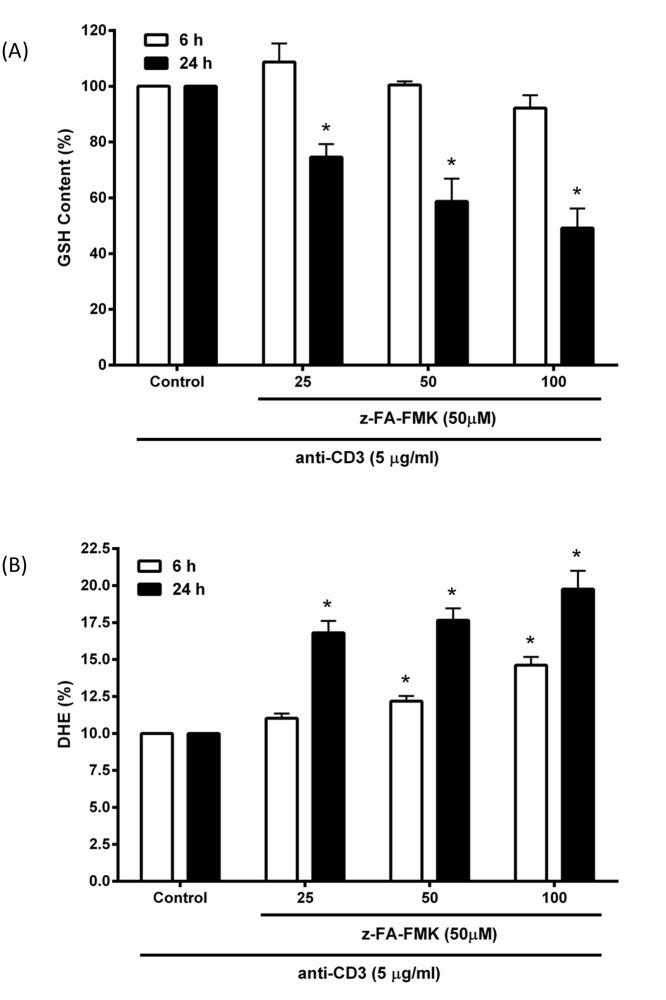
Effect of z-FA-FMK on intracellular GSH and ROS levels in activated primary T cells. PBMCs (1 x 10^6^/ml) were stimulated with anti-CD3 (5 μg/ml) in the presence of various concentrations of z-FA-FMK. The level of GSH (A) and ROS (B) was measured after 6 and 24 h using the fluorescent dye MCB and the DHE probe, respectively as described in Materials and Methods. The results for GSH are based on 1 x 10^4^ cells per sample and all results are means ± SEM from three separate donors. *,Significantly different (p < 0.01) from control with anti-CD3 alone.

It is well established that reduced levels of intracellular GSH is linked to the increase in ROS levels and diminished T cell proliferation [19–22]. Furthermore, we recently showed that z-VAD-FMK inhibits anti-CD3-induced T cell proliferation via oxidative stress trough the depletion of GSH [23]. We therefore examined whether oxidative stress may be responsible for the immunosuppressive effects induced by z-FA-FMK in primary T cells. To this end, the intracellular GSH and ROS levels were determined in anti-CD3-activated T cells in the presence or absence of z-FA-FMK. As illustrated in Fig 2A, z-FA-FMK at varying concentrations (25–100 μM) had little effect on the intracellular GSH levels in activated primary T cells after 6 h compared to control cells. However, after 24 h, there was a significant dose-dependent decrease in the intracellular GSH in anti-CD3-activated T cells (p < 0.05). These results suggest that z-FA-FMK is capable of depleting intracellular GSH in anti-CD3-activated T cells. Since reduced intracellular GSH is associated with an increased in ROS [19–22], the effect of z-FA-FMK on ROS generation was assessed using DHE dye which selectively detects intracellular superoxide anion (• O2–) production [24–26]. As shown in Fig 2B, z-FA-FMK at 25 μM has little effect on ROS levels in anti-CD3-activated T cells after 6 h. However, at higher concentrations (50 and 100 μM) the ROS generated in activated T cells were significantly increased compared to control (p < 0.05). After 24 h, the level of ROS in anti-CD3-activated T cells was further increased by z-FA-FMK in a dose-dependent manner. The carrier solvent DMSO (> 0.1%) has no effect on the intracellular GSH and ROS levels (data not shown). Collectively, these results suggest that z-FA-FMK induced oxidative stress in anti-CD3-activated T cells through the depletion of GSH and ROS production.

### Effect of antioxidants on the suppression of primary T cell proliferation mediated by z-FA-FMK

**Fig 3:**
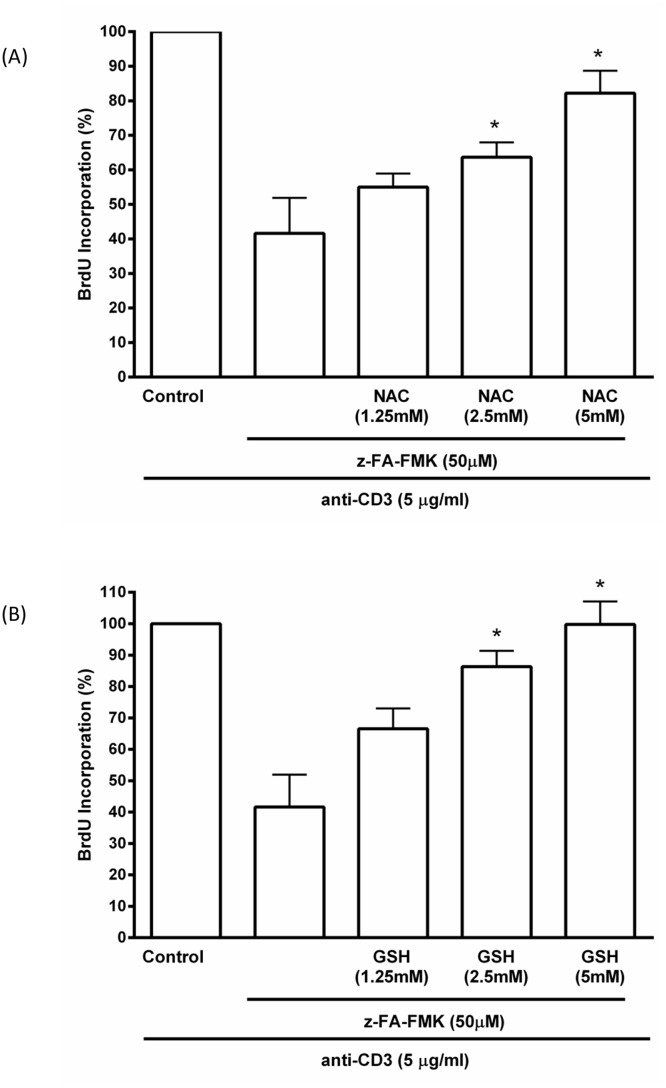
Effect of NAC and GSH on the inhibition of T cell proliferation mediated by z-FA-FMK. PBMCs (1 x 10^6^/ml) were stimulated with anti-CD3 (5 μg/ml) in the presence of 50 μM z-FA-FMK and varying concentrations of NAC (A) or GSH (B). BrdU incorporation was determined after 72 h as described in Materials and Methods. The relative incorporation of BrdU into control proliferation T cells (with anti-CD3 only) were normalised to 100%. Results are the mean ± SEM of at least three independent experiments. *,Significantly increased (p < 0.01) compared to z-FA-FMK + anti-CD3.

**Fig 4:**
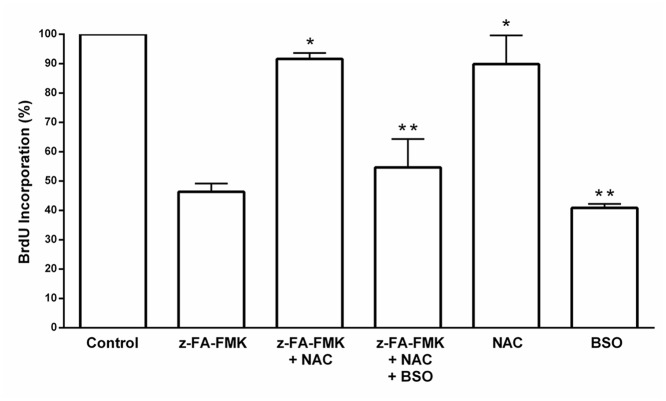
The effect of NAC on the inhibition of T cell proliferation mediated by z-FA-FMK is abolished by BSO. PBMCs (1 x 10^6^/ml) were stimulated with anti-CD3 (5 μg/ml) plus 50 μM z-FA-FMK in the presence or absence of NAC (5 mM) and BSO (0.5 mM) where indicated. BrdU incorporation was determined after 72 h as described in Materials and Methods. The relative incorporation of BrdU into control proliferation T cells (with anti-CD3 only) were normalised to 100%. Results are the mean ± SEM of at least three independent experiments. *,Significantly increased (p < 0.01) compared to z-FA-FMK; **Significantly decreased (p < 0.05) from z-FA-FMK + NAC.

**Fig 5:**
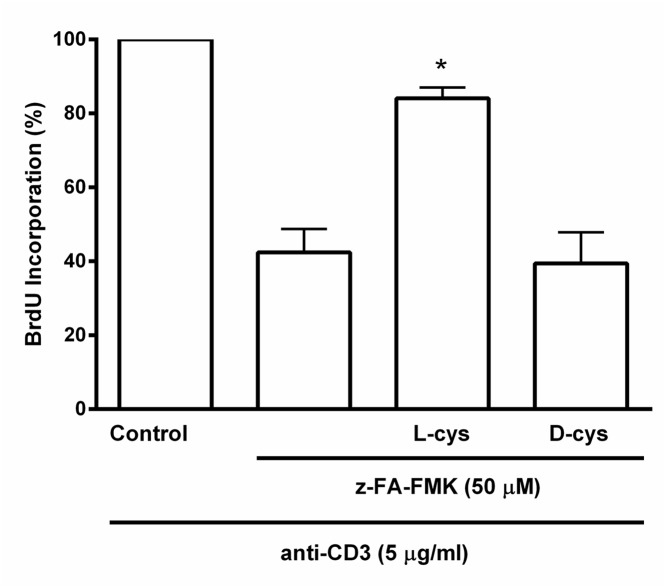
Effect of low molecular weight thiols on the inhibition of T cell proliferation mediated by z-FA-FMK. PBMCs (1 x 10^6^/ml) were stimulated with anti-CD3 (5 μg/ml) plus 50 μM z-FA-FMK in the presence or absence of low molecular weight thiols (5 mM) or BSO (0.5 mM) where indicated. BrdU incorporation was determined after 72 h as described in Materials and Methods. The relative incorporation of BrdU into control proliferation T cells (with anti-CD3 only) were normalised to 100%. Results are the mean ± SEM of at least three independent experiments. *,Significantly increased (p < 0.01) compared to z-FA-FMK + anti-CD3.

GSH is a major low molecular weight thiol in cells and is known to play an important role in T cell proliferation [27–29]. Its depletion suggests that the loss of intracellular GSH may be responsible for the inhibition of anti-CD3-induced T cell proliferation mediated by z-FA-FMK. We therefore examined whether the antioxidant, NAC could reverse the inhibition of T cell proliferation mediated by z-FA-FMK. As shown in Fig 3A, z-FA-FMK (50 μM) on its own inhibited anti-CD3-induced T cell proliferation and the presence of NAC readily abolished this inhibitory effect in a concentration-dependent manner (1.25–5 mM). Since NAC is a precursor of GSH biosynthesis we examined the effect of extracellular GSH on z-FA-FMK induced suppression of anti-CD3-mediated T cell proliferation. As illustrated in Fig 3B, GSH added exogenously fully restored the inhibition of T cell proliferation mediated by z-FA-FMK in a concentration-dependent manner (1.25–5 mM) suggesting that the depletion of intracellular GSH is the underlying mechanism of z-FA-FMK-induced immunosuppression. To further corroborate this, BSO, which irreversibly blocks the GSH synthesis pathway by inhibiting the enzyme γ-glutamylcysteine synthetase was added to anti-CD3-activated T cells co-treated with z-FA-FMK and NAC for 72 h [30]. As shown in Fig 4, NAC (5 mM) abolished the inhibition of T cell proliferation mediated by z-FA-FMK while the presence of BSO (0.5 mM) effectively blocked the ability of NAC to restore T cell proliferation. This clearly indicates that the ability of NAC to restore the inhibition of anti-CD3-induced T cell proliferation mediated by z-FA-FMK is via replenishing the intracellular pool of GSH. Since the inhibition of T cell proliferation mediated by z-FA-FMK was abolished by NAC and GSH, we further examined whether other low molecular weight thiols, such as L-cysteine and D-cysteine, would have the same effect. As illustrated in Fig 5, L-cysteine (5 mM), which is another precursor for GSH biosynthesis significantly restored (p < 0.05) the inhibition of T cell proliferation induced by z-FA-FMK, whereas D-cysteine which cannot be metabolised to GSH had little effect. Taken together, these results suggest that the immunosuppressive effects of z-FA-FMK are due to oxidative stress through the depletion of intracellular GSH.

### GSH restored z-FA-FMK-induced down-regulation of CD25 and CD69 in anti-CD3-activated T cells

**Fig 6:**
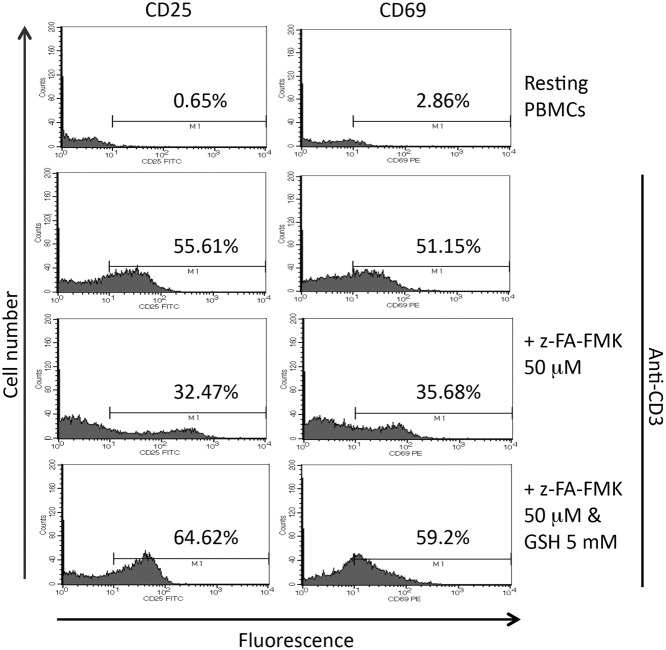
Effect of GSH on the inhibition of CD25 and CD69 expression in activated T cells mediated by z-FA-FMK. PBMCs (1 x 10^6^/ml) were stimulated with anti-CD3 (5 μg/ml) plus 50 μM z-FA-FMK in the presence or absence of 5mM GSH. After 72 h, the cells were stained with FITC-conjugated anti-CD25 or PE-conjugated anti-CD69 before analysis using flow cytometry as described in Materials and Methods. The results are one representative from three independent experiments.

Some of the earliest events that occur after T cell activation such as cytokine secretion and cell surface receptor up-regulation are inhibited by z-FA-FMK [11]. Since low molecular weight thiols were able to reverse the inhibition of T cell proliferation mediated by z-FA-FMK, we examined the effect of extracellular GSH on the expression of IL-2Rα (CD25) and the activated T cell marker, CD69. As shown in Fig 6, the percentage of cells that stained positive for CD25 and CD69 expression increased from around 0.65% and 3% in resting cells to 55.6% and 51.2% in activated T cells, respectively. Following treatment with z-FA-FMK (50 μM) the up-regulation of CD25 and CD69 in anti-CD3 activated T cells was reduced to 32.5% and 35.7%, respectively. In the presence of 5 mM GSH the down-regulation of CD25 and CD69 mediated by z-FA-FMK was completely restored to control levels. These results suggest that oxidative stress plays an important role in the down-regulation of CD25 and CD69 expression induced by z-FA-FMK in activated T cells.

### Effect of low molecular weight thiols on the inhibition of caspase-8 and caspase-3 processing mediated by z-FA-FMK in activated primary T cells

**Fig 7:**
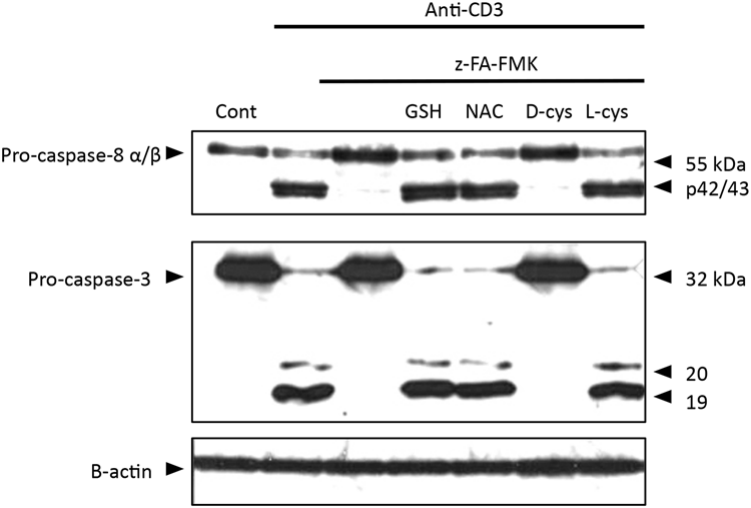
Effect of low molecular weight thiols on caspase-8 and -3 processing in z-FA-FMK-treated activated T cells. PBMCs (1 x 10^6^/ml) were stimulated with anti-CD3 (5 μg/ml) plus 50 μM z-FA-FMK in the presence or absence of antioxidants (5 mM). After 72 h, the cells were taken through a gradient density centrifugation using lymphoprep to obtain activated T cells. Whole cell lysates (20 μg protein) from activated T cells were resolved using 13% SDS-PAGE, transferred to nitrocellulose membrane and probed for caspase-8, caspase-3 and β-actin as described in Materials and Methods. The results are one representative of three independent experiments.

We have shown previously that z-FA-FMK completely blocks the processing of caspase-8 and caspase-3 to their subunits in activated T cells [11]. The finding that the low molecular weight thiols such as GSH, NAC and L-cysteine could reverse the inhibition of T cell proliferation as well as the expression of early T cell markers induced by z-FA-FMK raised the question whether these thiols could restore caspase-8 and caspase-3 processing in activated T cells in the presence of z-FA-FMK. In order to exclude the dying cells due to activation-induced cell death and growth factor deprivation, live activated T cells were purified using density gradient centrifugation (lymphoprep) prior to western blot analysis. As illustrated in Fig 7, the presence of z-FA-FMK (50 μM) completely blocked the activation and processing of caspase-8 and caspase-3, which is in good agreement with our previous studies [11]. The presence of 5 mM each of GSH, NAC and L-cysteine completely restored the inhibition of caspase-8 and caspase-3 processing mediated by z-FA-FMK in anti-CD3-activated T cells. The procaspase-8α and β were processed to the p42/43 fragments, respectively. Similar to control anti-CD3-activated T cells, caspase-3 was processed to the p20 and p17 fragments. As expected, D-cysteine which does not have any effect on the inhibition of T cell proliferation mediated by z-FA-FMK had little effect on the inhibition of caspase-8 and caspase-3 processing.

## Discussion

Accumulating evidence from a number of studies have now shown that z-FA-FMK is immunosuppressive and blocks LPS-induced cytokine secretion in macrophages [10], inhibits T cell activation and proliferation induced by mitogen as well as IL-2 in vitro [11]. These pleiotropic immunosuppressive effects may account for its remarkable therapeutic effect in suppressing the destruction of articular cartilage and bone in chronic inflammatory arthritis induced by adjuvant in mice [7–9]. In the present study we have examined the underlying mechanism involved in the inhibition of anti-CD3-induced T cell activation and proliferation mediated by z-FA-FMK.

In agreement with our earlier study [11], z-FA-FMK readily inhibited anti-CD3-induced T cell proliferation whereas z-FA-DMK which has a dimethyl group in the methylketone moiety was found to have little effect. In sharp contrast another analogue, z-FA-CMK, having a chlorine atom instead of a fluorine atom in the methylketone moiety was toxic and induced cell death in primary T cells. The toxicity of z-FA-CMK in primary T cells confirms our recent study where z-FA-CMK was found to induce both apoptosis and necrosis in Jurkat T cells [18]. It is well known that halomethylketone peptides having a chlorine atom instead of a fluorine atom are more reactive and can result in non-specific alkylation of proteins which may lead to their toxicity [3,31–33]. Since all three compounds blocked cathepsin B, it is unlikely that the inhibition of this enzyme play a role in the inhibition of proliferation and toxicity in primary T cells induced by z-FA-FMK and z-FA-CMK, respectively. It is also unlikely that the immunosuppression mediated by z-FA-FMK is caused by the FMK group alone, as other FMK-containing methylketone peptides such as z-YVAD-FMK and z-VRPR-FMK have little effect on anti-CD3-induced T cell proliferation. Interestingly, the N-terminal blocking group, benzyloxycarbonyl appears to play an important role in the immunosuppressive effects of z-FA-FMK since replacing it with biotin (biotin-FA-FMK) completely abrogated its ability to inhibit anti-CD3-induced T cell proliferation. Taken together, these results suggest that the immunosuppression mediated by z-FA-FMK is specific and requires a fluorine atom in the methylketone moiety and the benzyloxycarbonyl group at the N-terminal.

Although fluorometylketone peptides are not very reactive, they are capable of reacting with GSH directly as reported previously [31]. Furthermore, evidence from numerous studies have shown that human T lymphocytes depleted of intracellular GSH using pharmacological regulators [30,34–39] were unable to proliferate and accumulate high levels of ROS [19–21,40] We found that z-FA-FMK treatment leads to depletion of intracellular GSH levels in activated T cells with a concomitant increase in cellular ROS, suggesting that oxidative stress may be the underlying mechanism of z-FA-FMK immunosuppressive effect. Indeed, the inhibition of anti-CD3-induced T cell proliferation mediated by z-FA-FMK was abrogated by low molecular weight thiols such as GSH, NAC and L-cysteine. Both NAC and L-cysteine are precursors for GSH biosynthesis and is readily taken up by the cells. Once inside the cells, deacetylation of NAC will increase the intracellular cysteine levels, which is rate limiting in GSH biosynthesis [40]. Therefore, our results suggest that NAC and L-cysteine restores T cell proliferation in the presence of z-FA-FMK by acting as precursors for GSH biosynthesis. This is further corroborated when D-cysteine, which cannot be enzymatically converted to GSH, was unable to restore anti-CD3-induced T cell proliferation inhibited by z- FA-FMK. We observed that when cells were treated with BSO in addition to NAC and z-FA-FMK, NAC was ineffective and unable to restore T cell proliferation to control levels. Because BSO blocks γ-glutamylcysteine synthetase, the rate limiting step in GSH biosynthesis, low molecular weight thiols such as NAC and L-cysteine will not contribute much to GSH biosynthesis [30]. Our results also indicate that the depletion of intracellular GSH mediated by z-FA-FMK is not due to the inhibition of γ-glutamylcysteine synthetase otherwise both NAC and L-cysteine would not be able to restore the inhibition of anti-CD3-induced T cell proliferation. How z-FA-FMK diminishes intracellular GSH levels in activated T cells remains unclear. Although, peptidyl-FMKs have been reported to be capable of alkylating GSH directly in vitro [31], they are not very reactive toward GSH in vitro compared to CMKs. At physiological pH the rate of alkylation of GSH mediated by peptidyl FMKs is only 0.2% of the rate with peptidyl CMKs [31]. It remains to be determined whether this slow rate of GSH alkylation mediated by FMKs could result in the depletion of GSH in primary activated T cells over time. Collectively, our results and those published previously strongly implicate GSH as an important regulator of T cell proliferation and suggest a direct relationship between the availability of GSH and the proliferative response of T cells [19,21,41].

We and others have shown previously that z-FA-FMK also blocks NFκB signalling induced by antigen receptor stimulation, which in turn leads to the inhibition of IL-2 and IFN-γ production as well as the expression of CD25 [10,11]. We observed that the expression of CD25 and another early T cell activation marker, CD69 were inhibited by z-FA-FMK following primary T cell activation with anti-CD3. The expression of these two early T cell activation markers has been shown to be down-regulated following the depletion of GSH [28], suggesting that the depletion of intracellular GSH by z-FA-FMK may be responsible for the low expression of CD25 and CD69 in anti-CD3-stimulated T cells. Indeed, when endogenous GSH present, the inhibition of CD25 and CD69 expression mediated by z-FA-FMK following anti-CD3- induced T cell activation was completely restored. Taken together, our results suggest that intracellular GSH play a pivotal role in T cell activation and proliferation following antigen receptor stimulation.

Many studies in apoptosis research have used z-FA-FMK as a negative control for peptidyl-FMK caspase inhibitors because of its inertness towards caspases [42,43]. However, z-FA-FMK treatment effectively blocked both caspase-8 and caspase-3 processing in anti-CD3-activated T cells as shown in this study and previously [11]. Interestingly, GSH, NAC and L-cysteine were all able to fully restore the processing of caspase-8 and caspase-3 into their respective enzyme subunits. This suggests that oxidative stress through the depletion of intracellular GSH mediated by z-FA-FMK inhibits the activation and processing of caspases in activated T cells. However, it is well known that many toxicants induced apoptosis in various cell types including T cell lines via oxidative stress and the activation of caspases plays a central role [44,45]. An explanation that reconciles these seemingly contradictory observations is that the role played by caspases and its regulation in primary T cell activation and proliferation is different from apoptotic cell death. In primary T cells caspases are needed for cell proliferation following activation whereas caspases are needed for the execution of apoptosis leading to cleavage of cellular components and cell death. However, it was suggested previously that the activation of caspases in activated T cells, particularly caspase-3 was an artefact during cell lysis due to the use of detergents such as NP-40, deoxycholate and low concentration of SDS [46]. These detergents cause the release of granzyme B, which in turn activates caspase-3 and cleaved PARP, a caspase-3 substrate. It is unlikely that the activation of caspase-3 in our activated T cell model is due to extraction artefact for a number of reasons. Firstly, we used only Triton-X in our lysis buffer and not Nonidet P-40, deoxycholate or SDS which appears to be the cause of the artefact. Secondly, the activated T cells have no apoptotic characteristics or PARP cleavage even though caspase-3 was activated [47]. Finally, a number of studies have since been published demonstrating the activation of caspase-3 in activated T cells [48–50]. Collectively these evidences argue against the processing of caspase-3 in activated T cells as an extraction artefact. The lack of processing of caspase substrates, including PARP [11,49] in proliferating T cells suggest that the activity of the effector caspases may have been blocked by endogenous inhibitors like the inhibitor-of-apoptosis proteins (IAP) [51,52]. Indeed, previous study demonstrated that the caspase inhibitor X-linked inhibitor-of-apoptosis proteins (XIAP) interacts with cleaved caspase-3 and caspase-7 during human T cell proliferation, thereby blocking their full activation, substrate cleavage and cell death [52]. Another possibility is that the activated caspases are prevented from cleaving substrates that inevitably triggers apoptosis through limited subcellular compartmentalisation [52–54].

In summary, we have shown that the inhibition of anti-CD3-induced T cell activation and proliferation mediated by z-FA-FMK requires a fluorine atom in the methylketone moiety and the benzyloxycarbonyl group at the N-terminal. The immunosuppressive effect of z-FA-FMK is mediated through oxidative stress via the depletion of intracellular GSH, which is reversed by low molecular weight thiols such as GSH, NAC and L-cysteine but not D-cysteine, which cannot be metabolised into GSH. Furthermore, these thiols were able to restore the processing of caspase-8 and caspase-3 in z-FA-FMK treated T cells during activation and proliferation.
